# Decoding user satisfaction during reading of pre-defined question–answer pairs: BCI approach using delta and gamma EEG

**DOI:** 10.3389/fnins.2026.1934150

**Published:** 2026-09-30

**Authors:** Wanjoo Park, Salma Mansour, Laehyun Kim, Mohamad Eid

**Affiliations:** 1 Engineering Division, New York University Abu Dhabi, Abu Dhabi, United Arab Emirates; 2 Bionics Research Center, Korea Institute of Science and Technology (KIST), Seoul, Republic of Korea; 3 Department of HY-KIST Bio-convergence, Hanyang University, Seoul, Republic of Korea

**Keywords:** artificial intelligence, delta, EEG response, gamma, satisfaction

## Abstract

**Introduction:**

The rapid proliferation of large language models (LLMs) has intensified the need for robust evaluation frameworks to align artificial intelligence (AI) behavior with human expectations. While current reinforcement learning from human feedback (RLHF) strategies heavily rely on explicit, *post-hoc* self-reports, these metrics are fundamentally limited by subjective biases and user non-compliance.

**Methods:**

To address this, the current study presents a novel brain-computer interface (BCI) framework that systematically characterizes the continuous, implicit neural correlates of user satisfaction and dissatisfaction during a reading-based evaluative task involving pre-validated LLM-generated question-answer pairs. Sixty-four-channel electroencephalography (EEG) data were recorded from 30 participants while they read and evaluated various LLM-generated responses. To capture genuine subjective experiences, individual trials were relabeled based on post-trial 5-point Likert scale ratings into five distinct evaluative states, and spatiotemporal spectral analyses were performed. A ShallowFBCSPNet architecture was further used to evaluate single-trial classification between the very satisfied and very dissatisfied conditions under a subject-mixed cross-validation scheme.

**Results:**

Spatiotemporal spectral analyses revealed distinct neural oscillatory dynamics that temporally dissociate satisfaction and dissatisfaction. When reading highly satisfactory responses, a pronounced, sustained enhancement of low-frequency delta power was observed within the bilateral frontotemporal areas during the middle (4–8 s) and late (8–12 s) reading intervals, indexing high-order cognitive consolidation and reward processing. Conversely, processing highly dissatisfactory responses led to a localized suppression of high-frequency gamma power in the right frontotemporal region during the early interval (0–4 s), reflecting an immediate affective appraisal and expectation violation. Utilizing the ShallowFBCSPNet architecture, the system successfully discriminated between the “very satisfied” and “very dissatisfied” states, achieving an average accuracy of 81% under a subject-mixed cross-validation scheme.

**Discussion:**

These findings demonstrate that user evaluative states are encoded via distinct frequency-specific cortical networks over differing temporal windows. By bridging cognitive neuroscience and artificial intelligence, this study establishes the conceptual and empirical foundation for passive BCI-driven implicit feedback channels, laying groundwork toward future real-time paradigms for next-generation LLM alignment; the classification result should be interpreted as a feasibility demonstration rather than an estimate of real-world decoding accuracy.

## 1 Introduction

The rapid advancement of large language models (LLMs) has fundamentally transformed the landscape of human-computer interaction. Systems such as GPT-4, Claude, and Gemini have demonstrated remarkable capacity for natural language understanding and generation across a broad range of tasks, from question answering and summarization to creative writing and clinical decision support (Zhao et al., 2026). The deployment of these systems at scale has created an urgent need for mechanisms to evaluate response quality and align model behavior with human preferences and values (Dai et al., 2024). Central to this challenge is the collection of user feedback, which serves as the primary signal for iterative model improvement through techniques such as reinforcement learning from human feedback (RLHF) (Chaudhari et al., 2025) and direct preference optimisation (DPO) (Xiao et al., 2024).

Despite their importance, satisfaction and dissatisfaction are typically assessed through explicit self-report measures, including Likert-scale ratings, thumbs up/down judgements, and post-interaction questionnaires (Møller et al., 2024). While such methods are straightforward to deploy and interpret, they suffer from several fundamental limitations. Self-report measures are vulnerable to social desirability bias, whereby users provide responses they perceive to be expected rather than authentic reflections of their experience (Podsakoff et al., 2003). Retrospective evaluations are also susceptible to peak-end effects and recency bias, causing overall ratings to be disproportionately influenced by particularly salient moments rather than the complete interaction experience (Kahneman et al., 1993). Cognitive demands imposed by the interaction itself may further compromise the reliability of *post-hoc* judgements, particularly during lengthy or complex exchanges (Pilli and Nallur, 2026). Moreover, explicit feedback mechanisms depend on voluntary user participation and are therefore susceptible to self-selection bias, as users with particularly positive or negative experiences are disproportionately likely to provide ratings, potentially yielding a feedback signal that is not representative of the broader user population (Zhu et al., 2022). Consequently, explicit feedback mechanisms provide indirect, sparse, and potentially distorted representation of users' true evaluative states.

These limitations motivate the development of implicit, automatic methods capable of capturing satisfaction and dissatisfaction continuously and unobtrusively during interaction, without interrupting the user or relying on *post-hoc* recall. Physiological signals such as skin conductance, heart rate variability, and pupillometry have shown promise as indicators of cognitive and emotional engagement (Bell et al., 2018). However, these measures provide only indirect access to the cognitive processes underlying satisfaction and dissatisfaction judgements, which are fundamentally cortical in origin (Bell et al., 2018). Electroencephalography (EEG) offers a particularly promising alternative because it measures neural activity with millisecond temporal resolution while remaining relatively inexpensive, portable, and increasingly wearable (Portillo-Lara et al., 2021). These characteristics make EEG well suited for studying evaluative processes as they unfold during structured, reading-based human-AI evaluation tasks and for developing implicit feedback channels that can operate in real time.

In parallel, a rapidly growing body of work in 2025–2026 has begun to close the loop between LLMs and neural signal analysis more directly. Locally deployable LLM systems such as the EEG Emotion Copilot now demonstrate real-time interpretation of EEG-derived affective states together with automatic generation of interpretable reports (Chen et al., 2025), while standardized benchmarking frameworks such as LibEER have been introduced to enable rigorous, reproducible comparison of EEG-based affective decoding models (Liu et al., 2025). In a closely related vein, recent work has begun to decode users' cognitive and evaluative states directly during spoken dialogue with conversational AI systems (Zidar et al., 2026). These developments indicate that the present study's objective, establishing implicit EEG correlates of satisfaction with LLM-generated content, sits at the intersection of two converging fronts: EEG-based affective decoding increasingly powered by LLMs, and passive BCI paradigms increasingly extended toward direct human-AI interaction contexts.

Research on the neural basis of satisfaction initially emerged from information retrieval neuroscience. Using fMRI, Paisalnan et al. (2021) and Paisalnan et al. (2022) demonstrated that satisfaction arises from the interaction of affective and cognitive processes and is supported by a distributed network involving the insula, frontal cortex, and parietal regions, establishing satisfaction as a distinct cortical evaluative state. Building on this foundation, EEG studies began identifying neural markers associated with satisfaction-related evaluation. Pinkosova et al. (2022) and Pinkosova et al. (2023) reported ERP differences across varying levels of information relevance and showed that these responses are modulated by self-perceived knowledge, indicating that satisfaction-related neural activity is sensitive to cognitive context. At the oscillatory level, frontocentral delta power has emerged as a spectral index of positive outcome evaluation (Bernat et al., 2015), selectively enhanced following satisfying outcomes and absent during negative ones, while its valence effect has been shown to vary with cognitive demand (Qi et al., 2025), suggesting that satisfaction biomarkers are sensitive to individual cognitive context. Conversely, high-frequency gamma oscillations are consistently associated with negative valence and dissatisfaction, reflecting heightened affective arousal and increased cortical network integration across frontal and insular regions (Xiao et al., 2025).

Despite these advances, the neural mechanisms underlying satisfaction and dissatisfaction during interaction with conversational AI remain poorly understood. Existing work has identified satisfaction correlates in information retrieval paradigms with simple, externally cued stimuli (Paisalnan et al., 2022), which does not capture the higher-order cognitive demands of reading, comprehending, and evaluating a natural language response from a conversational AI. The present study addresses this gap by systematically characterizing the neural correlates of satisfaction and dissatisfaction elicited during the evaluation of LLM-generated responses, with the aim of advancing our understanding of the neural basis of human-AI interaction and contributing toward the development of implicit, cognitive, and real-time feedback mechanisms for personalized and aligned conversational AI systems.

## 2 Material and method

### 2.1 Participants and materials

A total of 30 participants were recruited for the EEG study. All participants provided informed consent before participation and were compensated. The study was approved by the Institutional Review Board of New York University Abu Dhabi (HRPP 2024–58). The inclusion criteria were 18–45 years old, right-handedness, and normal (or corrected-to-normal) vision/hearing. The exclusion criteria were a person with orthopedic hand conditions or a neurological or psychiatric disease history. All participants received monetary compensation for their participation in the study.

To construct experimental stimuli with reliable satisfaction and dissatisfaction labels, a separate online study was conducted prior to the EEG experiment. An initial pool of AI-generated stimuli was created, comprising 53 satisfactory question-answer pairs and 53 dissatisfactory question-answer pairs across 53 distinct questions. Ninety-six online participants aged 18–45 years old evaluated the materials by rating question interest and answer satisfaction on a five-point Likert scale. Only questions rated as highly interesting (ratings of 4 or 5) and answers that elicited strong satisfaction or dissatisfaction judgments (ratings of 4–5 or 1–2, respectively) were retained. Based on these ratings, the 25 highest-rated satisfactory and 25 highest-rated dissatisfactory question-answer pairs were selected as experimental stimuli for the EEG study. Because every question was paired with both a pre-validated satisfactory and a pre-validated dissatisfactory answer, stimulus content (question identity) was balanced across the intended valence conditions at the design stage.

### 2.2 Experimental setup and protocol

The experiment was conducted in a quiet laboratory environment with participants seated in front of a computer monitor, as shown in Figure 1. Visual stimuli were presented using Presentation (Neurobehavioral Systems, Inc.), which controlled stimulus timing, event markers, and behavioral response collection. Participants viewed a series of question-answer pairs and provided subjective satisfaction ratings using a five-button keypad. EEG data were continuously recorded throughout the experiment using a 64-channel EEG cap (Brain Products GmbH, Germany). Participants were instructed to minimize movement and maintain visual attention throughout the session to reduce motion-related artifacts.

**Figure 1:**
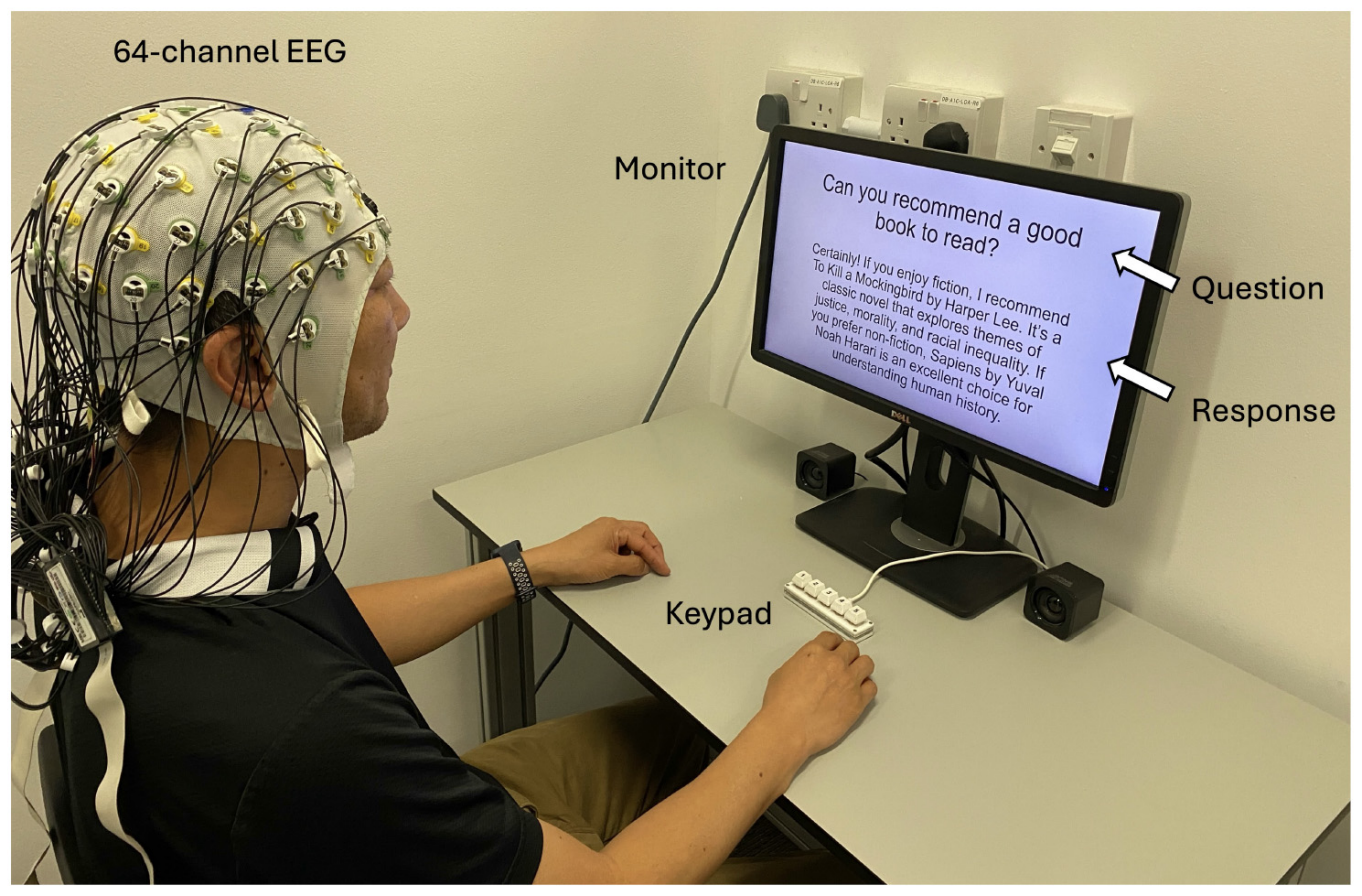
Experiment setup.

Participants were informed about the study procedure and completed a consent form. The experiment consisted of 50 trials, including 25 satisfactory and 25 dissatisfactory question-answer pairs presented in a predefined shuffled order. The stimuli were derived from the preliminary online study described earlier and were selected to maximize the contrast between satisfaction and dissatisfaction judgments.

Each trial followed the same structure shown in Figure 2. First, a fixation cross was presented for a randomized interval of either 1.5 s or 3 s to establish a baseline period. Next, a question was displayed on the screen for 5 s. Following question presentation, an answer was displayed for 12 s along with the question. Depending on the trial condition, the answer corresponded either to a satisfactory or a dissatisfactory response to the question. After reading the answer, participants provided a subjective evaluation using a five-point Likert scale ranging from 1 to 5: 1 (Very dissatisfactory), 2 (Dissatisfactory), 3 (Neutral), 4 (Satisfactory), and 5 (Very satisfactory). The rating screen remained visible until a response was recorded using the keypad. Throughout the experiment, event markers were transmitted at the onset of the question presentation, answer presentation, and participant rating response to facilitate subsequent EEG analysis.

**Figure 2:**
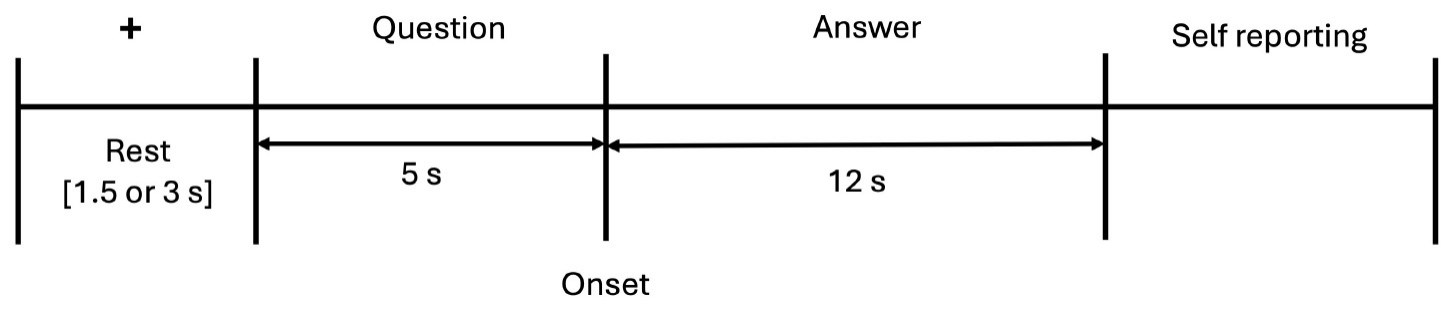
Experimental protocol.

### 2.3 EEG acquisition and preprocessing

EEG data were acquired using a BrainAmps amplifier (Brain Products GmbH, Germany) at a sampling frequency of 1 kHz. A 64-channel EEG cap (EasyCap, Herrsching, Germany), arranged according to the international 10–20 placement system, was utilized. The ground electrode was positioned at FPz, and the online reference electrode was located at FCz. To ensure high-quality recordings, the impedance of each electrode was maintained below 10 kΩ.

Initially, each trial, consisting of a question-answer pair, was assigned a predefined binary label (satisfactory or dissatisfactory). However, during the self-reporting phase of the experiment, participants explicitly evaluated their subjective level of satisfaction with each response using a 5-point Likert scale. Because capturing the participants' genuine, subjective experience was the primary focus of this study, we relabeled the trials based on these individualized ratings rather than the predefined categories. Consequently, the dataset was restructured into five distinct conditions reflecting the participants' actual responses: very dissatisfied, dissatisfied, neutral, satisfied, and very satisfied. Because the final very satisfied/very dissatisfied pools were defined by each participant's own post-trial rating rather than the predefined stimulus category, a given item could contribute to different perceptual categories across participants, reflecting genuine inter-individual evaluation rather than a fixed stimulus manipulation. Both participant- and item-level variability are therefore represented within each analyzed condition.

EEG data preprocessing was conducted offline using MATLAB release 2025b (MathWorks, United States) and the EEGLAB toolbox (v2024.2.1) (Delorme and Makeig, 2004). To reduce signal contamination arising from slow drifts and environmental noise, the EEG data were band-pass filtered between 0.1 and 85 Hz using a Hamming-windowed sinc finite impulse response filter. Line noise was further attenuated using a 50 Hz notch filter (bandwidth, 49.5–50.5 Hz). To enable robust artifact correction and reduce within-subject variability caused by physiological artifacts, artifact subspace reconstruction (ASR) was applied (Chang et al., 2019). The specific parameter configuration was set as follows: a flatline criterion of 5 s, a high-pass filter transition band of [0.25, 0.75] Hz, a minimum channel correlation threshold of 0.8, a line-noise criterion of 4 standard deviations, an ASR burst reconstruction standard deviation cutoff of *k* = 20, and a bad window rejection criterion of 25%. Subsequently, the data were re-referenced using a common average reference. Independent Component Analysis was not applied following ASR, as manual or semi-automated component classification risks discarding genuine cortical signal or retaining residual artifact, introducing experimenter-dependent variability. We therefore relied on a single, fully automated, conservatively parameterized ASR pipeline to minimize subjective component-selection bias. This ASR-centered, no-ICA approach follows established caution against aggressive artifact rejection discarding genuine cortical signal (Delorme, 2023), and matches a comparable pipeline previously validated by our group on EEG studies (Park et al., 2024, 2021). ASR performance is sensitive to the standard-deviation cutoff parameter (k) and is more effective at removing low-frequency (e.g., ocular) than high-frequency (e.g., myogenic) contamination (Miyakoshi, 2023); the specific, conservatively chosen parameters are reported above for transparency. The continuous EEG data were then segmented into epochs spanning from 8.5 s before to 14.5 s after the onset of each answer event. Baseline correction was performed in the time domain using a –6 s to –5 s baseline window before the onset. This is the rest period with fixation before the question period. The extended baseline interval was selected to better accommodate the high variability and potential confounding factors associated with dynamic cognitive brain activation during the question period, thereby enabling a more robust estimation of event-related changes while minimizing the influence of ongoing visual input. Electrodes FT9, FT10, TP9, and TP10 were excluded from further analysis due to their proximity to non-cortical regions and susceptibility to muscle-related artifacts. EEG data were then transformed into the time-frequency domain using 3-cycle Morlet wavelet transformation applied across the entire epoch (–8.5 to +14.5 s relative to answer onset). Power at each time-frequency point was subsequently expressed in decibels relative to the mean power within a 1-s defined baseline (–6 to –5 s), corresponding to the fixation rest period preceding the question.

Prior to statistical analysis, the normality of the data distribution was evaluated using the Jarque–Bera test. Given that the experimental design involved within-subject comparisons where the same participants evaluated both conditions, dependent statistical analyses were employed. Accordingly, a parametric paired *t*-test was utilized for data adhering to a normal distribution, whereas the non-parametric Wilcoxon signed-rank test was applied when the normality assumption was violated. Furthermore, because the 12-second answer presentation period was partitioned into three intervals for EEG analysis, resulting in three distinct comparisons, false discovery rate (FDR) correction was performed to adjust for multiple comparisons across three time-window comparisons. Furthermore, effect sizes for the statistical comparisons were evaluated using paired Cohen's *d*.

### 2.4 Single-trial EEG classification

To evaluate, as a proof-of-concept, whether the identified spectral differences carry sufficient single-trial information, we implemented a single-trial classification utilizing the ShallowFBCSPNet architecture (Schirrmeister et al., 2017). The model was trained to discriminate between the very satisfied and very dissatisfied conditions. The binary classification analysis was restricted to the very satisfied and very dissatisfied conditions, yielding a pooled dataset of 796 trials across all participants (379 very satisfied; 417 very dissatisfied), corresponding to approximately 26 trials per participant on average. In each cross-validation fold, trials were allocated as 64% for training, 16% for internal validation (used for early stopping), and 20% for held-out testing. Given the marginal 52/48 class imbalance, per-fold class weights were dynamically computed from the training labels to adjust the cross-entropy loss; additional resampling was not deemed necessary. Training and Hyperparameters: The model was optimized with a batch size of 256, a learning rate of 0.0005, a weight decay of 0.0005, and a dropout probability of 0.2. To ensure robust evaluation, data were split using 5-fold stratified cross-validation at the trial level, i.e., a subject-mixed (rather than subject-independent) validation scheme, in which trials from all 30 participants were pooled prior to splitting. Preprocessing and Augmentation: Per-channel z-score normalization statistics were computed solely from the training set and subsequently applied to the validation and test sets. To address the class imbalance between the two conditions, a weighted cross-entropy loss was computed per fold based on the training labels. Furthermore, to improve model generalization, four EEG-specific data augmentations (Gaussian noise, channel dropout, smooth time masking, and FT-surrogate phase randomization) were applied exclusively to the training data loader.

## 3 Results

### 3.1 Subjective evaluation

To ensure the validity of the experimental stimuli, we first verified whether the pre-selected satisfactory and dissatisfactory question-answer pairs successfully induced the intended psychological states of satisfaction and dissatisfaction among the participants. Figure 3 illustrates the participants' behavioral satisfaction ratings across both conditions. The evaluation was based on a five-point Likert scale: 1 (Very dissatisfactory) to 5 (Very satisfactory). For the satisfactory response condition, participants reported a high level of satisfaction, with a mean rating of 4.24 ± 0.37 (*M*± SD). Conversely, the dissatisfactory response condition yielded a significantly lower satisfaction rating of 1.73 ± 0.39. A paired-samples *t*-test revealed a highly significant statistical difference between the two conditions (Paired-samples *t*-test, *p* < 0.001). Additionally, the analysis revealed a paired Cohen's *d* of 5.27, indicating an exceptionally large effect size. These behavioral results robustly confirm that the experimental manipulation of satisfactory and dissatisfactory conditions was effectively established and perceived by the participants.

**Figure 3:**
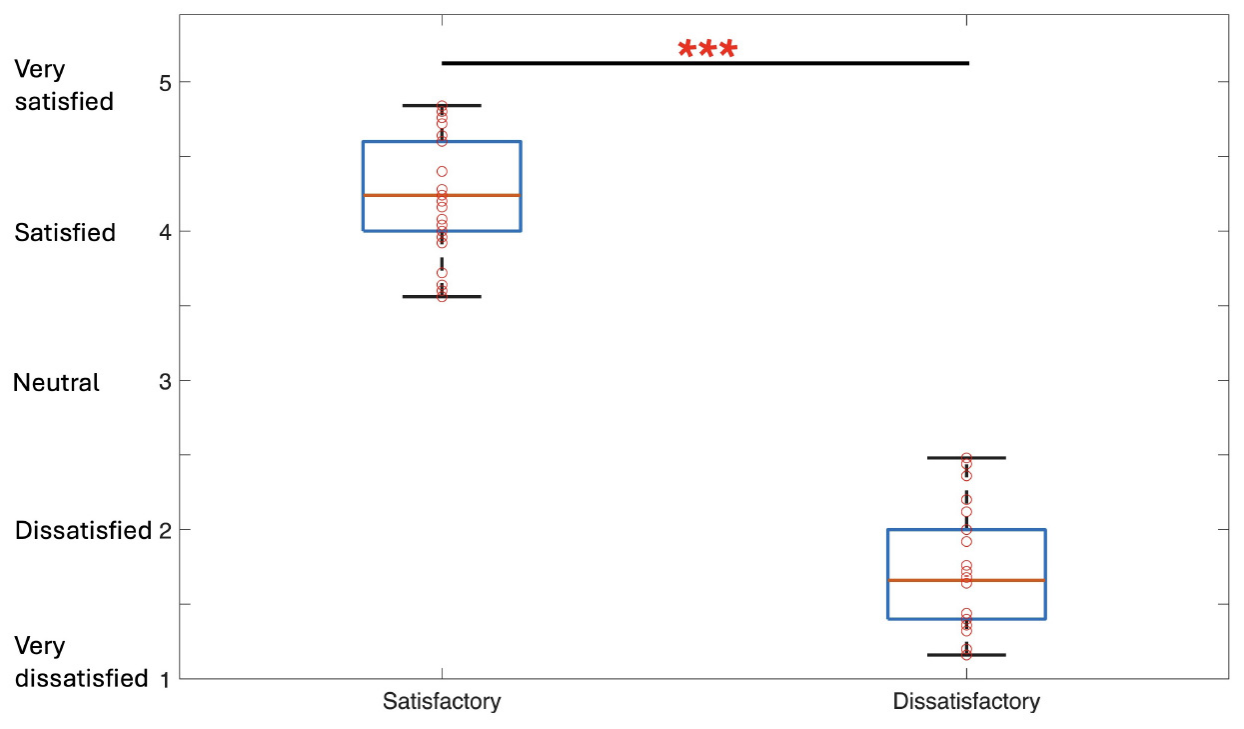
Subjective evaluation of answers. 5-point Likert scale for the pre-selected satisfactory and dissatisfactory question-answer conditions. Paired-samples *t*-test,^***^p < 0.001. Paired Cohen's *d* = 5.27.

### 3.2 EEG analysis

To investigate the neural alterations associated with response evaluation, we analyzed the power spectral density (PSD) distributions across the delta (1–4 Hz), theta (4–7 Hz), alpha (8–12 Hz), beta (13–32 Hz), gamma (33–49 Hz), and high-gamma (51–80 Hz) frequency bands. Specifically, we examined the frequency bands that exhibited prominent PSD changes during the 12-second answer presentation window relative to the baseline period. To define the regions of interest (ROIs) in an unbiased manner, the data from the satisfactory and dissatisfactory conditions were collapsed to identify cortical regions and frequency bands showing task-related alterations independent of the specific evaluation condition.

As illustrated in Figure 4, the delta band exhibited a distinct PSD increase in the bilateral frontotemporal areas, specifically encompassing the FT7, FC5, F7, FT8, FC6, and F8 channels, alongside a concurrent decrease in the prefrontal region (Fp1, Fp2, AF7, AF3, AFz, AF4, and AF8). Conversely, the gamma band demonstrated a localized PSD reduction prominently in the right frontotemporal area, including the F4, F6, F8, FC4, FC6, and FT8 channels. To establish an objective criterion for ROI definition, only channels demonstrating a mean PSD alteration of ±1.5 dB or greater relative to the baseline were included. Based on quantitative thresholds and distinct topographical patterns, these bilateral frontotemporal regions and prefrontal region for the delta band and the right frontotemporal region for the gamma band were formally selected as the ROIs for subsequent statistical evaluations.

**Figure 4:**
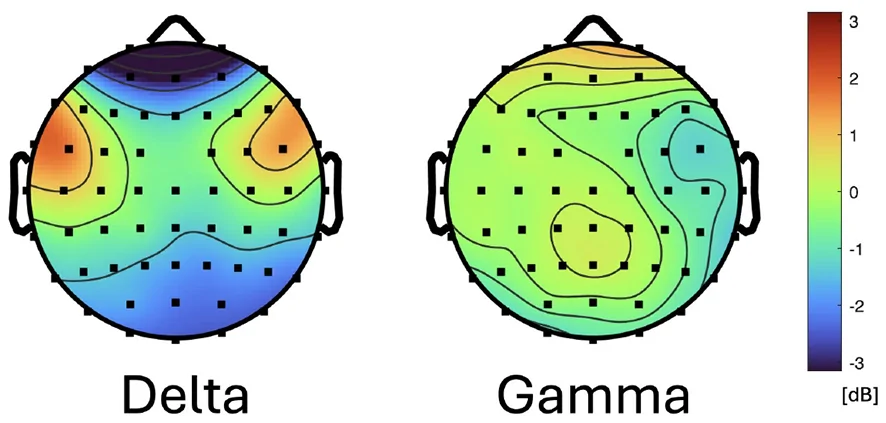
Topographical distributions of delta and gamma power spectral density collapsed across all evaluative conditions to ensure unbiased ROI selection during the 12-second answer period.

We aimed to investigate the participants' neural responses when receiving satisfactory versus dissatisfactory responses from the AI. Critically, because user satisfaction can vary substantially even for identical answers depending on individual backgrounds or subjective expectations, we stratified the EEG data into five distinct categories based on each participant's trial-by-trial 5-point Likert scale ratings. To maximize the contrast of the evaluative states, we specifically contrasted the individual subjective extremes: the very satisfied and very dissatisfied conditions.

The topographical maps in Figure 5 illustrate the spatial distributions of the delta and gamma PSD for these two evaluative states. For the delta band (Figure 5a), a subtle decrease in prefrontal delta PSD was observed during the question presentation phase. Furthermore, upon the appearance of the answer, a profound suppression of prefrontal delta PSD occurred across both the very satisfied and very dissatisfied conditions. This parallel trend indicated that prefrontal delta power did not serve as a reliable neural feature for discriminating satisfaction levels. Conversely, the subsequent enhancement of delta power within the bilateral frontotemporal areas was markedly more pronounced in the very satisfied condition compared to the very dissatisfied condition.

**Figure 5:**
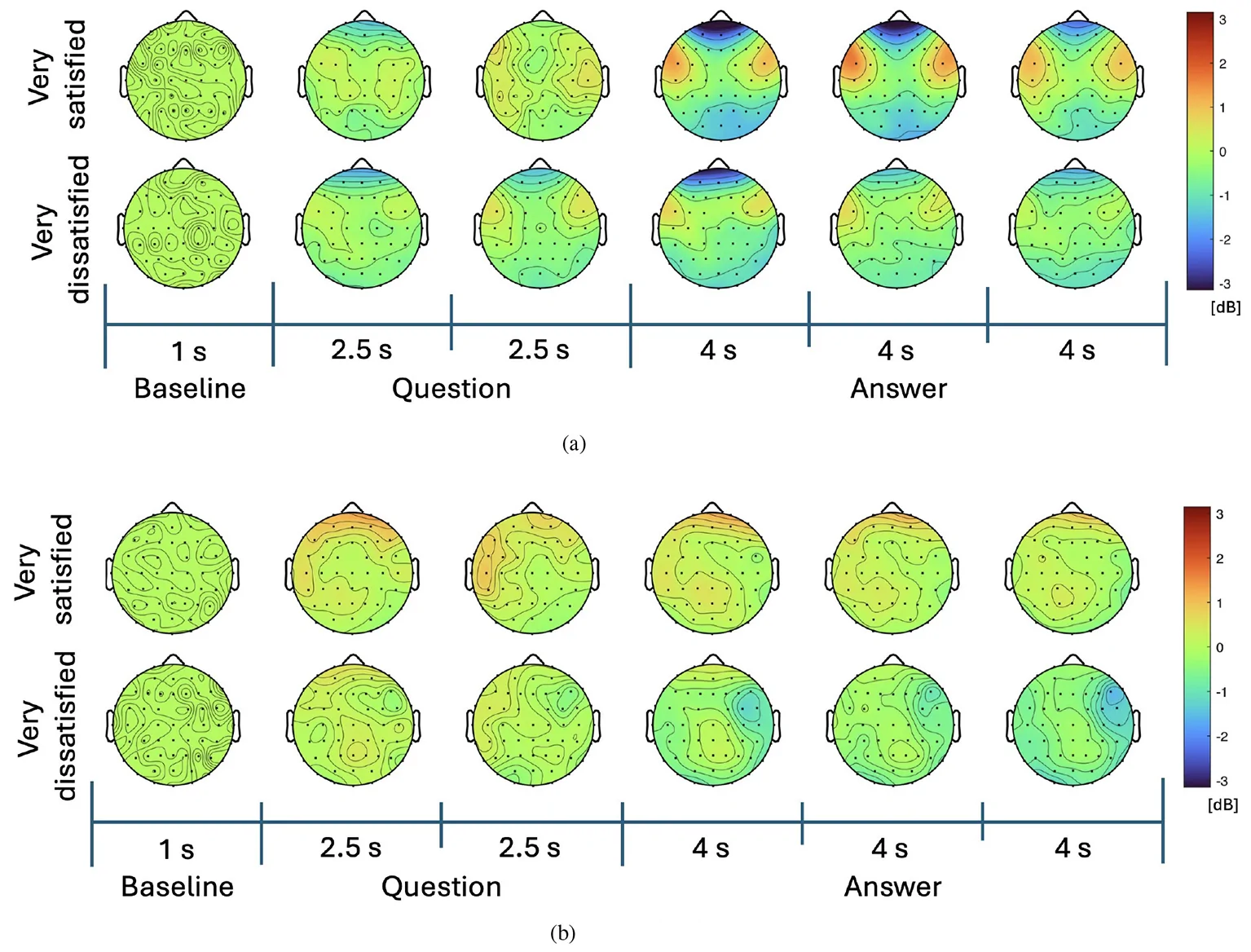
Topographical distributions of neural activity are compared between the very satisfied and very dissatisfied conditions. The 5-second question period is partitioned into two intervals and the 12-second answer period is partitioned into early (0–4 s), middle (4–8 s), and late (8–12 s) intervals for subsequent analyses. **(a)** Delta band. **(b)** Gamma band.

Regarding the gamma band (Figure 5b), the very satisfied condition elicited a transient increase in prefrontal gamma power, a phenomenon that was entirely absent in the very dissatisfied condition. Additionally, a distinct suppression of gamma PSD within the right frontotemporal region was observed exclusively while participants were reading responses they deemed very dissatisfactory.

Figure 6 presents the time-frequency spectrograms for both the delta and gamma bands. Specifically, Figure 6a displays the low-frequency characteristics within the bilateral frontotemporal areas. At the onset of the answer presentation, a transient increase in power was observed within both the delta and theta frequency bands. To systematically track the temporal dynamics of the subsequent evaluative processes, the 12-second answer-reading window was partitioned into three consecutive 4-second intervals: early (0–4 s), middle (4–8 s), and late (8–12 s). A closer examination of the 12-second interval during the very satisfied condition revealed a substantial, sustained elevation in delta PSD starting approximately 2 seconds post-onset. Statistical comparisons across the designated windows demonstrated that delta PSD was significantly higher in the very satisfied condition compared to the very dissatisfied condition during both the middle (4–8 s) and late (8–12 s) intervals (Wilcoxon signed-rank test, FDR-corrected, *p* < 0.05), as illustrated by the box plots in Figure 7. Additionally, the analysis yielded paired Cohen's *d* values of 0.46 and 0.47, respectively, indicating a medium effect size.

**Figure 7:**
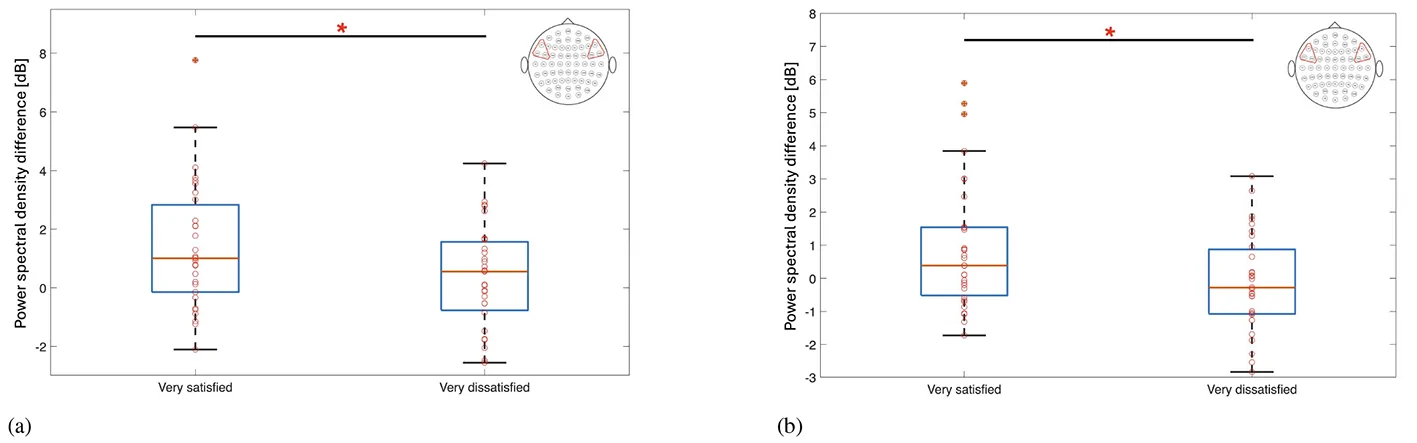
Statistical comparison of delta power spectral densities between very satisfied and very dissatisfied conditions. **(a)** Delta power spectral density difference during the middle (4–8 s) of answer period. Wilcoxon signed-rank test, FDR-corrected,^*^p < 0.05. Paired Cohen's *d* = 0.46. **(b)** Delta power spectral density difference during the last (8–12 s) of answer period. Wilcoxon signed-rank test, FDR-corrected,^*^p < 0.05. Paired Cohen's *d* = 0.47.

**Figure 6:**
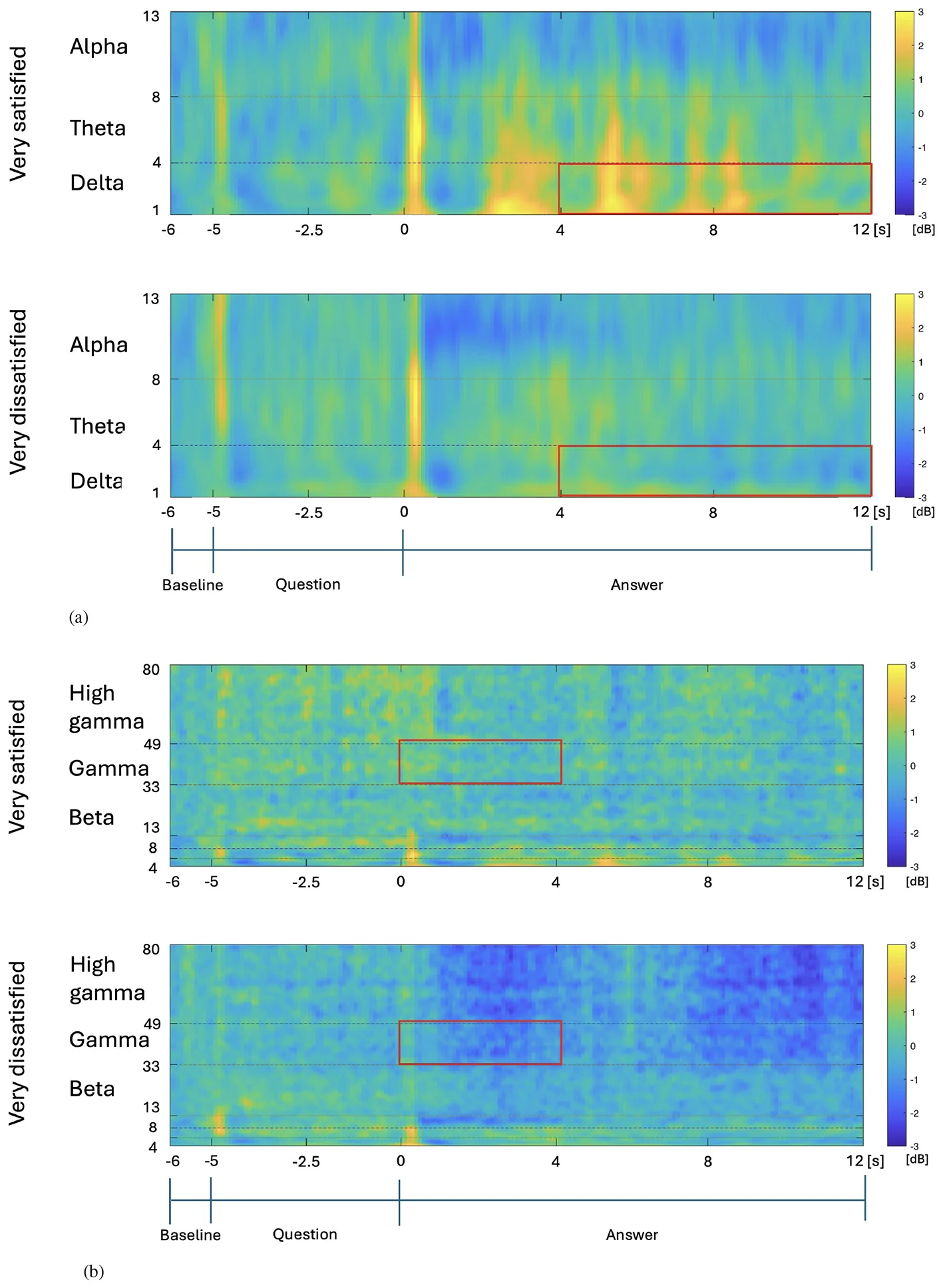
Condition-averaged time-frequency spectrograms. Red boxes indicate significant differences between very satisfied and very dissatisfied conditions. **(a)** Spectrogram of low-frequency bands in the bilateral frontotemporal area (FT7, FC5, F7, FT8, FC6, and F8). **(b)** Spectrogram of high-frequency bands in the right frontotemporal area (F4, F6, F8, FC4, FC6, and FT8).

The high-frequency PSD within the right frontotemporal area is depicted in Figure 6b. When participants processed very dissatisfied responses, a noticeable reduction in both gamma and high-gamma PSD was observed. As shown in Figure 8, the gamma PSD during the early 4-second interval was significantly attenuated in the very dissatisfied condition relative to the very satisfied condition (paired *t*-test, FDR-corrected, *p* < 0.05). Additionally, the analysis yielded a paired Cohen's *d* of 0.49, indicating a medium effect size. Although the mean gamma power in the late interval (8–12 s) also exhibited a descriptive reduction for the very dissatisfied condition, this difference did not achieve statistical significance. Furthermore, while the high-gamma band displayed a temporal pattern reminiscent of the gamma band, the differences between the two evaluative conditions did not reach the statistical threshold.

**Figure 8:**
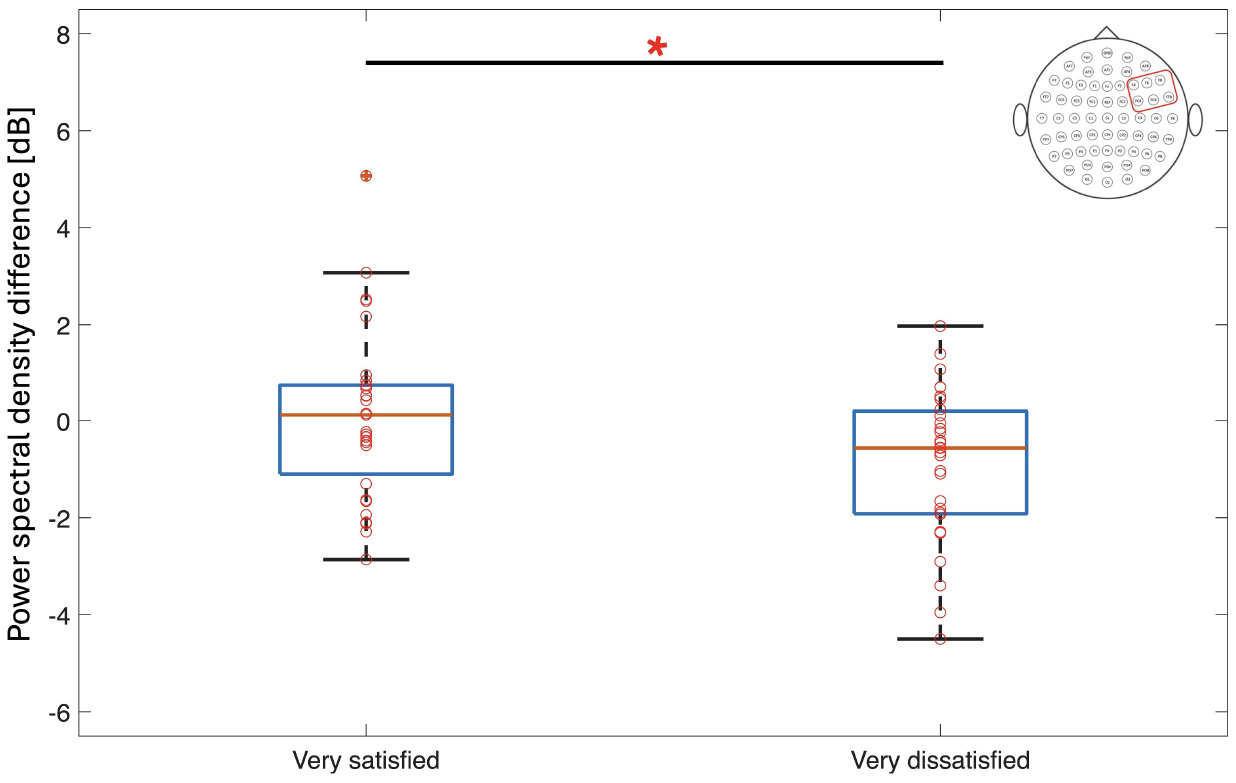
Statistical comparison of gamma power spectral density between very satisfied and very dissatisfied conditions during the early (0–4 s) of answer period. Paired *t*-test, FDR-corrected,^*^p < 0.05. Paired Cohen's *d* = 0.49.

### 3.3 Single-trial classification

The single-trial classification between the very satisfied and very dissatisfied conditions using the ShallowFBCSPNet model yielded an average accuracy of 0.81 ± 0.12 (M ± SD) across folds. Detailed per-class performance metrics (Table 1) indicated a highly balanced decoding capability. For the very satisfied class, it achieved a precision of 0.8016, a recall of 0.7995, and an F1-score of 0.8005. For the very dissatisfied class, the model achieved a precision of 0.8182, a recall of 0.8201, and an F1-score of 0.8192. As reflected in the confusion matrix (Table 2), the true positive rates were balanced, with 79.9% of very satisfied trials and 82.0% of very dissatisfied trials correctly classified. These results provide preliminary evidence that the spectro-spatial differences carry discriminative single-trial information, under a subject-mixed cross-validation scheme.

**Table 1:** Classification report.

Class	Precision	Recall	F1-score
Very satisfied	0.8016	0.7995	0.8005
Very dissatisfied	0.8182	0.8201	0.8192

**Table 2:** Confusion matrix.

		Predicted label	
		Very satisfied	Very dissatisfied
**True label**	Very satisfied	79.9%	20.1%
	Very dissatisfied	18.0%	82.0%

## 4 Discussion

The current study provides the first systematic characterization of the continuous, unprompted neural correlates of user satisfaction and dissatisfaction during reading-based evaluation of LLM-generated textual responses. Our behavioral findings validated that the experimental stimuli successfully induced distinct evaluative states. Crucially, EEG recordings revealed that these states are underpinned by distinct neural oscillatory dynamics across the delta and gamma frequency bands, reflecting a complex interplay between cognitive appraisal and affective processing as the human-AI interaction unfolds over time. This temporal and spectral divergence highlights that cortical responses to identical external stimuli are substantially modulated by an individual's intrinsic preference and psychological traits, echoing prior findings in personalized neuro-evaluation (Ha et al., 2021).

A major finding of this study is the pronounced enhancement of delta power within the bilateral frontotemporal areas during the middle (4–8 s) and late (8–12 s) intervals of reading satisfactory responses. Low-frequency delta oscillations have been traditionally associated with the processing of feedback valence and reward processing in decision-making paradigms (Bernat et al., 2015). In the context of conversational AI, this frontotemporal delta activation may reflect the progressive realization of satisfaction as the user comprehends a highly relevant and fulfilling response, aligning with recent evidence that delta oscillations code feedback valence under varying cognitive demands (Qi et al., 2025).

Conversely, we observed a profound suppression of prefrontal delta power across both the very satisfied and very dissatisfied conditions immediately following answer presentation. It is worth noting that the direction of this effect argues against a residual ocular-artifact origin. Blink- and saccade-related contamination is characterized by large-amplitude, low-frequency power increases at frontopolar sites due to the corneo-retinal dipole (Croft and Barry, 2000), whereas the observed prefrontal delta pattern was a sustained attenuation relative to baseline in both conditions. Because ocular artifacts would be expected to elevate, not suppress, prefrontal delta power, and both conditions underwent identical ASR-based preprocessing (Chang et al., 2019), a residual-artifact account is unlikely to fully explain this pattern. Nevertheless, trial-by-trial blink rate was not independently quantified in this study, and future work incorporating dedicated EOG channels would allow this possibility to be excluded more directly.

This parallel trend indicates that prefrontal delta activity is decoupled from subjective satisfaction ratings and instead indexes the shared higher-order cognitive demands of text reading, comprehension, and structural evaluation required in conversational paradigms, distinguishing it from simpler information retrieval tasks (Paisalnan et al., 2022; Pinkosova et al., 2023). Therefore, while prefrontal regions manage the baseline cognitive load of interacting with an LLM, the bilateral frontotemporal delta power serves as a selective, localized biomarker for genuine user satisfaction.

In contrast to the late-emerging delta patterns, high-frequency gamma band oscillations demonstrated distinct alterations in the early phase (0–4 s) of response evaluation. Specifically, processing very dissatisfied responses led to a significant, localized suppression of gamma power in the right frontotemporal region. High-frequency gamma activity is widely implicated in cortical network integration and affective arousal during emotional or evaluative tasks (Xiao et al., 2025). Furthermore, previous neuroergonomic studies have demonstrated that high-frequency gamma oscillations serve as a sensitive neural index for decoding explicit user preference profiles across interactive hardware interfaces (Park et al., 2018). The early attenuation of right frontotemporal gamma power observed here may represent an implicit neural marker of expectation violation or immediate negative appraisal when a user encounters a suboptimal or irrelevant AI response. Given that the frequency range of cranial electromyographic (EMG) activity heavily overlaps with cortical gamma-band oscillations, particularly in the vicinity of the frontalis and temporalis muscles (Hipp and Siegel, 2013; Muthukumaraswamy, 2013), one might suspect that the observed alterations could be confounded by myogenic artifacts. However, several lines of evidence strongly counter this artifactual interpretation. Peripheral muscle contamination triggered by negative emotional expressions (e.g., frowning or jaw clenching during dissatisfaction) typically manifests bilaterally or symmetrically across frontotemporal channels. In the present study, no comparable gamma power fluctuations were observed in the left frontotemporal region under identical conditions, which supports a neurogenic rather than a myogenic origin for the right-sided suppression. Furthermore, this right-lateralized topographical specificity aligns with existing neuroergonomic literature indicating that user preferences and hedonic evaluations are selectively modulated within right frontal and frontotemporal gamma networks (Park et al., 2018). Taken together, these factors confirm that the observed right frontotemporal gamma dynamics genuinely reflect internal cognitive-affective evaluation rather than peripheral muscular noise. Furthermore, the transient increase in prefrontal gamma power observed exclusively in the very satisfied condition suggests an early-stage engagement of executive or attentional networks when a response meets or exceeds user expectations (Herrmann et al., 2004). Together with the delta band results, these findings indicate a temporal dissociation in user evaluation: an early affective appraisal mediated by high-frequency gamma dynamics is followed by a sustained cognitive consolidation of satisfaction indexed by low-frequency frontotemporal delta power.

Beyond the group-level spectro-temporal differences, the present study demonstrated the practical viability of decoding these evaluative states on a single-trial basis. By employing a ShallowFBCSPNet model, we achieved an 0.81 ± 0.12 classification accuracy between the very satisfied and very dissatisfied conditions. Importantly, the balanced precision and recall rates indicate that the model did not simply bias toward a majority class, but successfully learned the underlying neural representations of user satisfaction. The implementation of robust EEG-specific data augmentations (e.g., FT-surrogate phase randomization and time masking) mitigated overfitting, ensuring that the model generalizes well across trials. This high decoding performance effectively bridges the gap between theoretical neurophysiological findings and practical BCI applications. It is important to note that this accuracy was obtained under a subject-mixed, rather than subject-independent, scheme, and should therefore be interpreted as a proof-of-concept demonstration of feasibility rather than an estimate of real-world decoding accuracy.

The identification of robust neural markers for satisfaction and dissatisfaction has profound implications for the future of human-AI alignment. Current alignment strategies, such as reinforcement learning from human feedback (RLHF) and direct preference optimization (DPO), depend heavily on explicit preference labels (Dai et al., 2024; Xiao et al., 2024; Chaudhari et al., 2025). However, these explicit feedback loops are fundamentally limited by user compliance, self-selection biases, and retrospective memory distortions (Kahneman et al., 1993; Podsakoff et al., 2003; Møller et al., 2024). By establishing frontotemporal delta power and right frontotemporal gamma power as reliable neural indices, our work lays the conceptual foundation for passive, brain-computer interface (BCI)-driven feedback channels. This direction parallels emerging efforts to extend passive BCI paradigms directly into human-AI interaction contexts. For instance, EEG-specific work has recently demonstrated the feasibility of decoding cognitive load and implicit agreement during spoken dialogue with conversational AI systems (Zidar et al., 2026), lending independent support to the broader premise that mental-state decoding can be transferred from controlled laboratory tasks to naturalistic human-AI exchanges. Furthermore, the growing availability of LLM-assisted EEG interpretation tools, such as the EEG Emotion Copilot, which couples real-time affective EEG decoding with automatic natural-language report generation (Chen et al., 2025), alongside standardized benchmarking resources such as LibEER (Liu et al., 2025), suggests a plausible near-term technical pathway by which the delta- and gamma-band biomarkers identified here could be integrated into a fully closed-loop, LLM-interpretable pipeline, in which neural evaluative signals are decoded, summarized in natural language, and fed back into the alignment process with minimal human intervention. While an empirical optimization framework has been successfully leveraged to maximize user comfort and subjective pleasantness in sensory hardware applications, such as vibrotactile alarm systems designed for hedonic awakening (Korres et al., 2018), the current study expands this user-centric alignment paradigm into the high-order domain of semantic and textual AI interactions. Incorporating implicit neural signals into the alignment pipeline could enable continuous, real-time model calibration without interrupting the user's cognitive flow, mitigating the distortions inherent in traditional explicit metrics (Bell et al., 2018). This approach marks a significant step toward developing highly adaptive, personalized conversational AI systems that align directly with the user's internal cognitive and affective states.

Despite the promise of these findings, several limitations must be acknowledged. First, the experiment was conducted in a controlled laboratory environment using a high-density, tethered 64-channel EEG cap, which limits ecological validity. While high-density setups are critical for identifying robust ROIs and spectral features, practical deployment in real-world human-AI interaction will require lightweight, wireless, and wearable BCI technologies (Portillo-Lara et al., 2021). Future research should explore the feasibility of decoding user satisfaction using sparse electrode configurations optimized for these frontotemporal regions. Second, the sample size of the present study was relatively limited, which may constrain the generalizability of the identified neural biomarkers to a broader and more diverse population. Additionally, while the question-answer pairs were strictly controlled for interest and quality, future studies should investigate neural dynamics during more open-ended, multi-turn dialogues where cognitive load and satisfaction levels evolve dynamically over extended periods. Third, the single-trial classification employed a trial-level, subject-mixed cross-validation scheme, in which trials from the same participant could appear in both training and test folds within a given iteration. While this maximized the training data available given our sample size, it does not isolate purely subject-independent generalization, and individual differences in EEG topology may have partially contributed to the reported accuracy. Future work using leave-one-subject-out cross-validation will be necessary to establish generalization to unseen individuals. Fourth, neither ICA nor separate EOG channel recording was performed; although the direction of the observed prefrontal delta suppression (an attenuation rather than the elevation typical of ocular contamination) argues against an artifactual origin, this remains an indirect inference. Direct prefrontal EOG recording could facilitate a clearer interpretation of prefrontal delta power.

## 5 Conclusions

This study successfully demonstrates the feasibility of decoding implicit user satisfaction and dissatisfaction during reading-based evaluation of pre-defined, LLM-generated question–answer pairs using EEG. Our findings establish that subjective user evaluation of complex LLM textual responses is not a monolithic event but a temporally and spectrally dissociable process. Cognitive satisfaction is characterized by a late-emerging, sustained enhancement of frontotemporal delta power, while dissatisfaction is registered via an early, transient suppression of right frontotemporal gamma activity. By validating that subjective preferences significantly modulate cortical dynamics during natural text reading, this work overcomes the inherent constraints of traditional retrospective self-reports. More importantly, these localized spatiotemporal biomarkers lay the empirical groundwork for integrating passive BCI systems into the loop of machine learning alignment pipelines (e.g., RLHF or DPO). Utilizing continuous, implicit neural feedback will allow future conversational systems to calibrate themselves dynamically to a user's internal cognitive and affective states without interrupting the natural flow of interaction. Ultimately, this integration represents a transformative step toward deeply personalized, adaptive, and human-centric artificial intelligence. Furthermore, as a proof-of-concept demonstration, single-trial classification using a ShallowFBCSPNet achieved an 81% accuracy under a subject-mixed cross-validation scheme. While this offline result indicates that the delta and gamma biomarkers carry single-trial discriminative information, establishing genuine subject-independent, real-time decoding will require future work using leave-one-subject-out validation on larger cohorts.

## Data Availability

The raw data supporting the conclusions of this article will be made available by the authors, without undue reservation.

## References

[B1] Bell L. Vogt J. Willemse C. Routledge T. Butler L. T. Sakaki M. (2018). Beyond self-report: a review of physiological and neuroscientific methods to investigate consumer behavior. Front. Psychol. 9:1655. doi: 10.3389/fpsyg.2018.01655 PMC6137131 30245657

[B2] Bernat E. M. Nelson L. D. Baskin-Sommers A. R. (2015). Time-frequency theta and delta measures index separable components of feedback processing in a gambling task. Psychophysiology 52, 626–637. doi: 10.1111/psyp.12390 PMC4398588 25581491

[B3] Chang C.-Y. Hsu S.-H. Pion-Tonachini L. Jung T.-P. (2019). Evaluation of artifact subspace reconstruction for automatic artifact components removal in multi-channel EEG recordings. IEEE Trans. Biomed. Eng. 67, 1114–1121. doi: 10.1109/TBME.2019.2930186 31329105

[B4] Chaudhari S. Aggarwal P. Murahari V. Rajpurohit T. Kalyan A. Narasimhan K. . (2025). Rlhf deciphered: a critical analysis of reinforcement learning from human feedback for llms. ACM Comput. Surv. 58, 1–37. doi: 10.1145/3743127

[B5] Chen H. Zeng W. Chen C. Cai L. Wang F. Shi Y. . (2025). EEG emotion copilot: Optimizing lightweight llms for emotional EEG interpretation with assisted medical record generation. Neural Netw. 192:107848. doi: 10.1016/j.neunet.2025.107848 40683189

[B6] Croft R. J. Barry R. J. (2000). Removal of ocular artifact from the EEG: a review. Clin. Neurophysiol. 30, 5–19. doi: 10.1016/S0987-7053(00)00055-1 10740792

[B7] Dai J. Pan X. Sun R. Ji J. Xu X. Liu M. . (2024). “Safe rlhf: safe reinforcement learning from human feedback,” in International conference on learning representations, 50750–50777.

[B8] Delorme A. (2023). EEG is better left alone. Sci. Rep. 13:2372. doi: 10.1038/s41598-023-27528-0 PMC9911389 36759667

[B9] Delorme A. Makeig S. (2004). EEGlab: an open source toolbox for analysis of single-trial EEG dynamics including independent component analysis. J. Neurosci. Methods 134, 9–21. doi: 10.1016/j.jneumeth.2003.10.009 15102499

[B10] Ha J. Park W. Park S. I. Im C.-H. Kim L. (2021). EEG response to game-craving according to personal preference for games. Soc. Cogn. Affect. Neurosci. 16, 995–1005. doi: 10.1093/scan/nsaa131 PMC8421702 33064824

[B11] Herrmann C. S. Munk M. H. Engel A. K. (2004). Cognitive functions of gamma-band activity: memory match and utilization. Trends Cogn. Sci. 8, 347–355. doi: 10.1016/j.tics.2004.06.006 15335461

[B12] Hipp J. F. Siegel M. (2013). Dissociating neuronal gamma-band activity from cranial and ocular muscle activity in EEG. Front. Hum. Neurosci. 7:338. doi: 10.3389/fnhum.2013.00338 PMC3706727 23847508

[B13] Kahneman D. Fredrickson B. L. Schreiber C. A. Redelmeier D. A. (1993). When more pain is preferred to less: adding a better end. Psychol. Sci. 4, 401–405. doi: 10.1111/j.1467-9280.1993.tb00589.x

[B14] Korres G. Jensen C. B. F. Park W. Bartsch C. Eid M. (2018). A vibrotactile alarm system for pleasant awakening. IEEE Trans. Haptics 11, 357–366. doi: 10.1109/TOH.2018.2804952 29994371

[B15] Liu H. Yang S. Zhang Y. Wang M. Gong F. Xie C. . (2025). Libeer: a comprehensive benchmark and algorithm library for EEG-based emotion recognition. IEEE Trans. Affect. Comput. 16, 3596–3613. doi: 10.1109/TAFFC.2025.3605833

[B16] Miyakoshi M. (2023). Artifact subspace reconstruction: a candidate for a dream solution for EEG studies, sleep or awake. Sleep 46:zsad241. doi: 10.1093/sleep/zsad241 PMC10710985 37715954

[B17] Møller C. G. Ang K. E. de Lourdes Bongiovanni M. Khalid M. S. Wu J. (2024). “Metrics of success: evaluating user satisfaction in ai chatbots,” in Proceedings of the 2024 8th international conference on advances in artificial intelligence, 168–173. doi: 10.1145/3704137.3704182

[B18] Muthukumaraswamy S. D. (2013). High-frequency brain activity and muscle artifacts in meg/EEG: a review and recommendations. Front. Hum. Neurosci. 7:138. doi: 10.3389/fnhum.2013.00138 PMC3625857 23596409

[B19] Paisalnan S. Moshfeghi Y. Pollick F. (2021). “Neural correlates of realisation of satisfaction in a successful search process,” in Proceedings of the association for information science and technology, 282–291. doi: 10.1002/pra2.456

[B20] Paisalnan S. Pollick F. Moshfeghi Y. (2022). “Neural correlates of satisfaction of an information need,” in International conference on machine learning, optimization, and data science (Springer), 443–457. doi: 10.1007/978-3-031-25891-6_34

[B21] Park W. Alsuradi H. Eid M. (2024). EEG correlates to perceived urgency elicited by vibration stimulation of the upper body. Sci. Rep. 14:14267. doi: 10.1038/s41598-024-65289-6 PMC11189896 38902337

[B22] Park W. Kim D.-H. Kim S.-P. Lee J.-H. Kim L. (2018). Gamma EEG correlates of haptic preferences for a dial interface. IEEE Access 6, 22324–22331. doi: 10.1109/ACCESS.2018.2827023

[B23] Park W. Kim S.-P. Eid M. (2021). Neural coding of vibration intensity. Front. Neurosci. 15:682113. doi: 10.3389/fnins.2021.682113 PMC8631937 34858124

[B24] Pilli S. Nallur V. (2026). “Emulating aggregate human choice behavior and biases with gpt conversational agents,” in Proceedings of the 2026 CHI conference on human factors in computing systems, 1–37. doi: 10.1145/3772318.3791835

[B25] Pinkosova Z. McGeown W. J. Moshfeghi Y. (2022). “Revisiting neurological aspects of relevance: an EEG study,” in International conference on machine learning, optimization, and data science (Springer), 549–563. doi: 10.1007/978-3-031-25891-6_41

[B26] Pinkosova Z. McGeown W. J. Moshfeghi Y. (2023). Moderating effects of self-perceived knowledge in a relevance assessment task: an EEG study. Comput. Hum. Behav. Rep. 11:100295. doi: 10.1016/j.chbr.2023.100295

[B27] Podsakoff P. M. MacKenzie S. B. Lee J.-Y. Podsakoff N. P. (2003). Common method biases in behavioral research: a critical review of the literature and recommended remedies. J. Appl. Psychol. 88:879. doi: 10.1037/0021-9010.88.5.879 14516251

[B28] Portillo-Lara R. Tahirbegi B. Chapman C. A. Goding J. A. Green R. A. (2021). Mind the gap: state-of-the-art technologies and applications for EEG-based brain-computer interfaces. APL Bioeng. 5:031507. doi: 10.1063/5.0047237 PMC8294859 34327294

[B29] Qi C. Zhu J. Chen L. Tong L. Jia S. (2025). Perceptual load modulates the delta oscillation and the contribution of delta oscillation to reward positivity during feedback valence encoding. Front. Psychol. 16:1658756. doi: 10.3389/fpsyg.2025.1658756 PMC12394510 40893861

[B30] Schirrmeister R. T. Springenberg J. T. Fiederer L. D. J. Glasstetter M. Eggensperger K. Tangermann M. . (2017). Deep learning with convolutional neural networks for EEG decoding and visualization. Hum. Brain Mapp. 38, 5391–5420. doi: 10.1002/hbm.23730 PMC5655781 28782865

[B31] Xiao S. Youssef N. Zhang Q. Lin X. Qiu Z. Liu W. . (2025). High gamma EEG responses to emotional stimuli in virtual reality: insights from local activation and distributed characteristics. Front. Hum. Neurosci. 19:1623331. doi: 10.3389/fnhum.2025.1623331 PMC12325198 40772244

[B32] Xiao T. Yuan Y. Zhu H. Li M. Honavar V. G. (2024). “Cal-dpo: calibrated direct preference optimization for language model alignment,” in Advances in neural information processing systems, 114289–114320. doi: 10.52202/079017-3630

[B33] Zhao W. X. Zhou K. Li J. Tang T. Dong Z. Hou Y. . (2026). A survey of large language models. Front. Comput. Sci. 20:2012627. doi: 10.1007/978-981-96-6259-3

[B34] Zhu Q. Lo L. Y.-H. Xia M. Chen Z. Ma X. (2022). “Bias-aware design for informed decisions: Raising awareness of self-selection bias in user ratings and reviews,” in Proceedings of the ACM on human-computer interaction, 1–31. doi: 10.1145/3555597

[B35] Zidar L. M. Wicke P. Bhatia P. Lutz R. Klug M. Zander T. O. (2026). “Decoding workload and agreement from EEG during spoken dialogue with conversational AI,” in 2026 14th international conference on brain-computer interface (BCI) (IEEE), 1–6. doi: 10.1109/BCI69045.2026.11435049

